# Lipoma of the middle finger: A case report and review of literature

**DOI:** 10.1097/MD.0000000000008309

**Published:** 2017-10-20

**Authors:** Zhongqing Hu, Zhenshuang Yue, Yanghua Tang, Yi Zhu

**Affiliations:** a Department of Orthopedics, Xiaoshan Traditional Chinese Medical Hospital; b The Second Affiliated Hospital of Zhejiang University, Hangzhou, Zhejiang, P.R. China.

**Keywords:** finger, lipoma, MRI, ultrasound

## Abstract

**Rationale:**

Lipomas originated from fingers are rare and the dissection is necessary when the lipomas limit the finger movement or cause pain.

**Patient concerns:**

A 57-year-old male was admitted to our department due to a painless swelling on the volar side of the middle finger of the right hand. The flexion movement of the distal interphalangeal joint was limited.

**Diagnoses:**

: Imaging studies and open biopsy confirmed that it was a finger lipoma.

**Interventions:**

: An excisional biopsy was performed.

**Outcomes:**

: The patient recovered completely after two weeks.

**Lessons:**

: Based on this case and literature we reviewed, ultrasound and MRI should be used to diagnose the finger lipoma and excision was the main treatment option.

## 1 Introduction

Lipomas are benign tumors occurring in regions of abundant adipose tissues. Previous study reported that hand lipomas were mainly found in the thenar and hypothenar regions.^[1]^ However, lipomas originated from fingers were rare, with a incidence of 1%.^[2]^ Clinically, most lipomas are asymptomatic mass with clear margin and moderate mobility. However, tumors that compress nearby nerves will cause pain. Here, we report a rare case of lipoma of the middle finger of the right hand, with no prior traumatic history.

## 2 Methods

The private information and medical records were obtained from patient with informed consent and with approval of the institutional ethics committee (Xiaoshan Traditional Chinese Medical Hospital).

## 3 Case report

A 57-year-old man arrived at our Hand Surgery Department, with a complaint of a painless swelling on the volar side of the middle finger of the right hand. The mass had been found for more than 7 years and was growing slowly.

**Figure 1:**
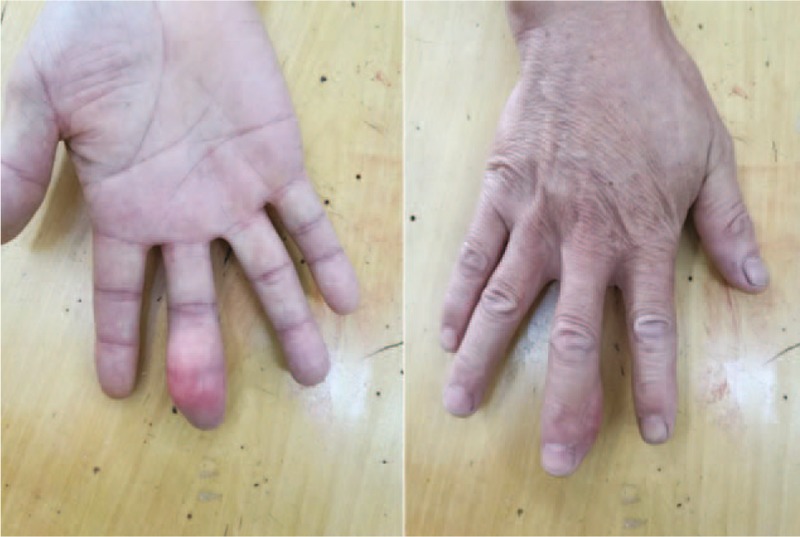
Preoperative appearance of the patient showing a third right hand finger volar tumor.

Physical examination showed a soft, mobile subcutaneous mass of size 35 mm × 15 mm localized at the middle and distal phalanxes and the overlying skin was normal. The flexion movement of the distal interphalangeal joint was limited now due to the swelling. The patient denied fingertip paresthesia, palpable pulse, fluctuation, thrill, or bruit (Fig. 1).

**Figure 2:**
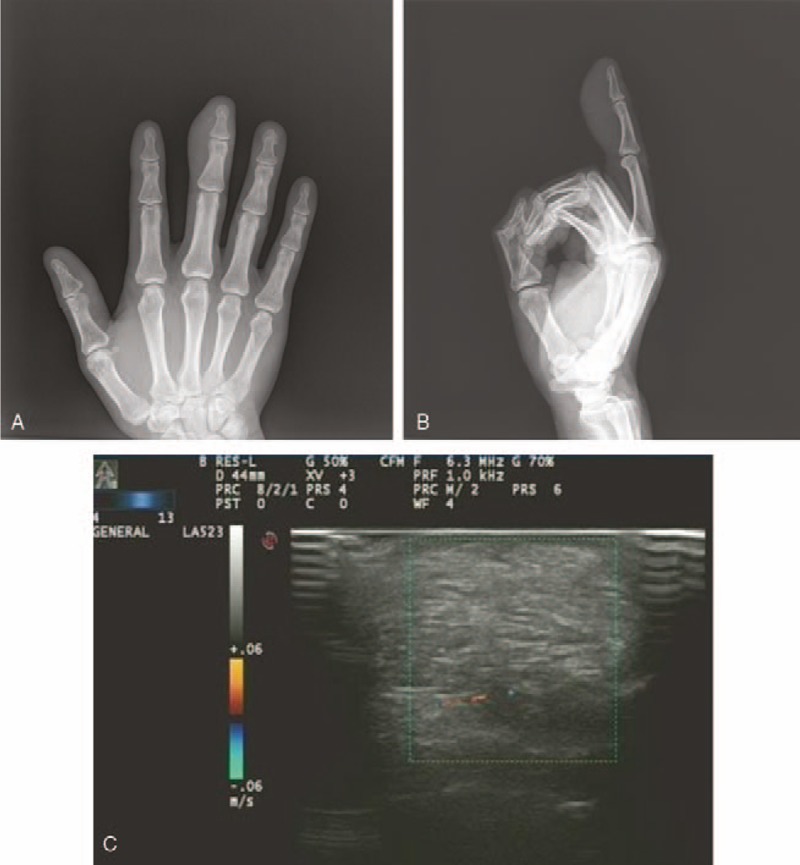
A: Radiography of the middle finger shows a soft tissue swelling of low density over the distal phalanx (anteroposterior view). B: Radiography of the middle finger shows a soft tissue swelling of low density over the distal phalanx (lateral view). C: An ultrasound scan shows a well-defined hyperechoic subcutaneous mass and no internal flow is detected.

The x-ray showed a soft tissue swelling without bone erosion or soft tissue calcification (Fig. 2A and B). An ultrasound scan showed a well-defined hyperechoic subcutaneous mass with scarce septations. No internal blood flow was detected by Doppler ultrasound (Fig. 2C).

**Figure 3:**
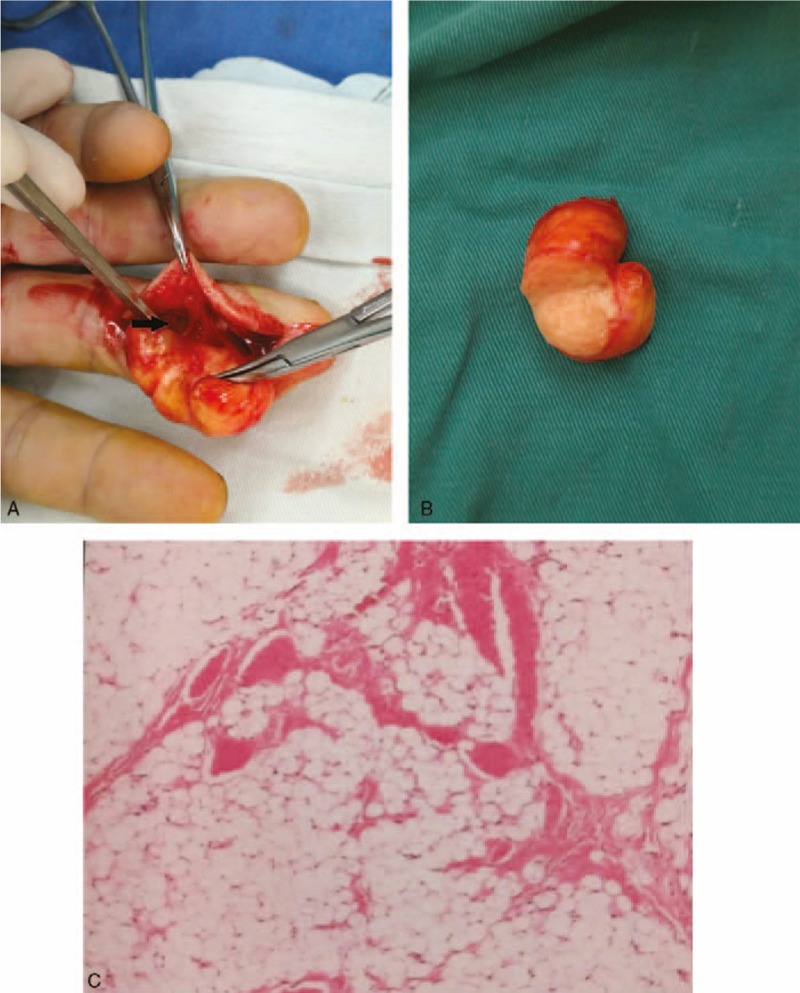
A: Tumor is nourished by one main pedicle (black arrow). B: Yellow fat-like tissue is involved. C: Histopathology shows mature fat cells (H&E stain ×200).

An excisional biopsy was performed. The shape of tumor was ovoid and the boundary was clear. It was nourished by 1 main pedicle that originated from ulnar digital bundle branch (Fig. 3A). The mass was cut into halves and yellow fat-like tissue was found (Fig. 3B). The pathological diagnosis showed it was a lipoma without neural component or malignant transformation (Fig. 3C).

## 4 Discussion

Lipomas account for approximately 16% of soft-tissue mesenchymal tumors.^[3]^ Previous study has showed that 36 cases of lipomas were identified since the first lipoma of the finger was reported in 1959.^[2,4]^ Most lipomas are asymptomatic. However, patients often feel uncomfortable once the tumors grow to a large size. In our case, the patient only presented with flexion limitation.

Etiology and pathogenesis of this disease are still unclear. Genetic, traumatic, and metabolic factors have been investigated and the conclusions were still inconsistent.^[3]^ The typical ultrasonography of lipomas shows a well-defined oval hyperechoic subcutaneous mass, with a thin capsule. In addition, no internal blood flow could be detected.^[5,6]^ X-ray should be performed to exclude bone erosions. Intraosseous lipomas are radiologically visualized as an osteolytic lesion and may be accompanied by bone expansion and cortical thinning.^[7]^ Especially, magnetic resonance imaging (MRI) was a better option to support the diagnosis of lipoma. In fact, researchers had found that MRI can provide a highly accurate diagnosis in more than 80% of the cases.^[8]^ Differential diagnosis based on imaging characteristics includes neoplastic and non-neoplastic lesions, such as giant cell tumor, implantation cyst, and spindle cell lipoma.^[9,10]^ Lipomas usually don’t require treatment. However, if the lipomas cause pain or restrict joint movement, surgical resection is recommended.

In our case, the finger lipoma was mainly detected by ultrasound. At last, the tumor was removed surgically and the patient recovered completely after 2 weeks.
